# Spatial capture–recapture with random thinning for unidentified encounters

**DOI:** 10.1002/ece3.7091

**Published:** 2020-12-08

**Authors:** José Jiménez, Ben C. Augustine, Daniel W. Linden, Richard B. Chandler, J. Andrew Royle

**Affiliations:** ^1^ Instituto de Investigación en Recursos Cinegéticos (IREC, CSIC‐UCLM‐JCCM) Ronda de Toledo, 12 Ciudad Real 13071 Spain; ^2^ U.S. Geological Survey John Wesley Powell Center Cornell Department of Natural Resources Ithaca New York 14853 USA; ^3^ Greater Atlantic Regional Fisheries Office NOAA National Marine Fisheries Service 55 Great Republic Drive Gloucester Massachusetts 01922 USA; ^4^ Warnell School of Forestry and Natural Resources University of Georgia 180 E. Green Street Athens Georgia 30602 USA; ^5^ U.S. Geological Survey Patuxent Wildlife Research Center 12100 Beech Forest Road Laurel Maryland 20708 USA

**Keywords:** brown bear, density estimation, noninvasive sampling, spatial capture–recapture, uncertain identity, unmarked, *Ursus arctos*

## Abstract

1. Spatial capture–recapture (SCR) models have increasingly been used as a basis for combining capture–recapture data types with variable levels of individual identity information to estimate population density and other demographic parameters. Recent examples are the unmarked SCR (or spatial count model), where no individual identities are available and spatial mark–resight (SMR) where individual identities are available for only a marked subset of the population. Currently lacking, though, is a model that allows unidentified samples to be combined with identified samples when there are no separate classes of “marked” and “unmarked” individuals and when the two sample types cannot be considered as arising from two independent observation models. This is a common scenario when using noninvasive sampling methods, for example, when analyzing data on identified and unidentified photographs or scats from the same sites.
2. Here we describe a “random thinning” SCR model that utilizes encounters of both known and unknown identity samples using a natural mechanistic dependence between samples arising from a single observation model. Our model was fitted in a Bayesian framework using NIMBLE.
3. We investigate the improvement in parameter estimates by including the unknown identity samples, which was notable (up to 79% more precise) in low‐density populations with a low rate of identified encounters. We then applied the random thinning SCR model to a noninvasive genetic sampling study of brown bear (*Ursus arctos*) density in Oriental Cantabrian Mountains (North Spain).
4. Our model can improve density estimation for noninvasive sampling studies for low‐density populations with low rates of individual identification, by making use of available data that might otherwise be discarded.

## Graphical abstract

Here we describe a “random thinning” spatial capture–recapture model that utilizes encounters of both known and unknown identity samples using a natural mechanistic dependence between samples arising from a single observation model and where individuals cannot be classed by mark status.

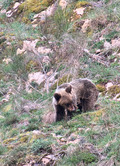

## 1 INTRODUCTION

The estimation of population size using capture–recapture models is a standard approach in wildlife research and provides a rigorous quantitative method for informing species conservation and management (Williams et al., 2002). Traditional sampling requires the physical capture and artificial marking of individuals over multiple surveys to create encounter histories. Capture–recapture models then use the encounter histories to estimate the probability of capture and, by extension, the proportion of the total population that was captured (Otis et al., 1978). A key requirement of this approach is the individual identification associated with each capture event. More recently, noninvasive sampling methods have made use of naturally occurring marks, whether in the form of distinguishing physical features that can be photographed (e.g., spot patterns) or DNA samples that can be passively collected and used to identify individuals. The increased application of noninvasive sampling can be attributed to many factors, including logistical conveniences of data collection, improvements in technology, and animal welfare concerns (Long et al., 2008). Noninvasive sampling has been a particularly useful approach for monitoring wide‐ranging mammals (e.g., carnivores), which occur at low densities and are otherwise difficult to physically capture over the large extents necessary for useful inferences.

One challenge associated with noninvasive sampling in the context of capture–recapture models is that natural marks, including genotypes, are often imperfectly observed. Photographs, especially from camera traps, may not provide a sufficiently clear and complete view of natural marks to verify individual identification for each encounter (Burton et al., 2015). Further, most wildlife species do not have individually unique features to allow for photo‐identification methods, limiting more widespread application of capture–recapture models to camera trapping. Noninvasive genetic techniques can be applied to any species that deposits genetic material (e.g., hair or scat), which can be collected to extract DNA and identify individual genotypes (Waits & Paetkau, 2005); however, genetic sampling has its own challenges for establishing the individual identity associated with each sample (Augustine et al., 2020; Augustine et al., 2019). The quantity and quality of DNA from noninvasive samples is typically very low, and extraction procedures often require extensive replication to improve genotyping accuracy (Taberlet et al., 1999). Environmental degradation can render any sample unusable for determining identity, either through a lack of DNA amplification or errors caused by contamination or random chance (Waits & Paetkau, 2005). Samples whose individual identities cannot be established with high confidence are typically discarded, which can often comprise a large portion of all samples collected. This data reduction has the effect of reducing the precision of population parameter estimates; thus, statistical methods that can utilize these discarded samples are desirable.

Substantial progress has been made using spatial capture–recapture (SCR) as the basis for jointly analyzing samples with known and unknown individual identities. Chandler and Royle (2013) first introduced an SCR model with fully latent individual identities that can be applied to a collection of samples with no individual identities at all, demonstrating that there is information about the individual identities of samples contained in their spatial location of capture that can be utilized to estimate population parameters. While the estimates from this model are frequently of very low precision, Chandler and Royle (2013) demonstrated that this method produced much better estimates when the set of unmarked samples is combined with a set of samples from marked individuals with known individual identities. This approach combining data from marked and unmarked individuals was later termed “spatial mark–resight” (SMR; Sollmann et al. (2013)), which has been quite successful for estimating population parameters for partially marked populations where a subset of individuals is either manually or naturally marked.

While SMR provides a framework for combining known and unknown identity samples, it is limited to the case where individuals can be separated into “marked” and “unmarked” classes and where “unmarked” individuals can never provide an individual identity. However, for noninvasive sampling and natural marks, it is often the case that although all individuals are identifiable, any individual can produce either individually identified or unidentified samples. Thus, all samples could theoretically be identifiable, but not all end up so. For example, individual identities in genetic capture–recapture are lost due to features of the individual DNA sample and the process of DNA amplification, not due to features of the individual. Further, when camera trapping a species where individuals are nearly equally identifiable (e.g., using flank patterns), identities are lost at the sample level due to poor animal orientation or photograph quality. For both remote cameras and genetic sampling, the level of resulting data loss can be substantial. For example, Kendall et al. (2016) identified 382 samples as grizzly bear (*Ursus arctos),* among which 127 (33.2%) were identified at individual level. Hooker et al. (2015) identified 9%–13% of hair samples from American black bear (*Ursus americanus)*; Murphy et al. (2018) identified ca. 7.8% of hair samples and ca. 16.1% of scat samples from coyote (*Canis latrans)*; Aziz et al. (2017) identified 24% for hair and scat samples—jointly—from tiger (*Panthera tigris*); Sun et al. (2017) identified 36% for hair from American black bears; and Murphy et al. (2016) identified 43.6% for hair from American black bears. In camera trap studies, the identification rate is rarely reported (Johansson et al., 2020), but Ngoprasert et al. (2012) described 2% of raw images that were identifiable in Asiatic black bears (*Ursus thibetanus*) and sun bears (*Helarctos malayanus*); Molina et al. (2017) identified 2.3% in Andean bear (*Tremarctos ornatus*), and Somers et al. (2018) identified 54% in leopard (*Panthera pardus*). This loss of information can be translated into lower precision in the estimates.

With the hypothesis that the use of all data could improve the precision of the estimates, we describe an SCR model that combines identified and unidentified samples from a single class of individuals (as opposed to “marked” and “unmarked” classification in SMR) using modified methods from Chandler & Royle (2013). The model allows for inference on abundance and distribution while accounting for uncertainty about the partially observed encounter histories. We demonstrate the performance of this model relative to using the identified encounter histories alone via simulation and apply it to a large‐scale noninvasive genetic sampling effort of brown bear (*Ursus arctos*) across a 2,624 km^2^ region of the Eastern Cantabrian Mountains, North Spain, where only 60% of the DNA samples provided an individual identity. We illustrate how noninvasive sampling studies can maximize the information used to provide population inferences from capture–recapture designs despite difficulties in determining individual identity.

## 2 METHODS

### 2.1 Model formulation

Our model is a standard SCR model, with an additional random thinning process that determines which samples' individual identities are observed. The standard SCR model assumes that individual activity centers i=1,2,⋯,N are distributed over a region or state space $\left(S\right)$ and individuals are exposed to sampling by some trap or detector array within S. The distribution of individuals activity centers $s_{i}=\left(s_{i_{1}},s_{i_{2}}\right)$ is typically described by a homogeneous point process, such that $s_{i}∼\mathrm{Uniform}\left(S\right)$, which we will adopt here. Inhomogeneous point processes can also be used to model variation in the distribution of individuals (Borchers & Efford, 2008; Royle et al., 2014). The activity centers are latent variables to be estimated by the model given the trap‐specific encounters for the n observed individuals at traps j=1,2,⋯J with locations $x_{j}=\left(x_{j_{1}},x_{j_{2}}\right)$. Assuming that encounter frequencies are Poisson‐distributed and a decreasing function of the distance $d_{\mathrm{ij}}$ between individual activity center $s_{i}$ and trap location $x_{j}$, the expected encounter rate can be specified as:

$$\lambda_{\mathrm{ij}}=\lambda \left(s_{i},x_{j}\right)=\lambda_{0}\exp\left(‐d_{\mathrm{ij}}^{2}/2\sigma^{2}\right)$$

Here, $\lambda_{0}$ is the expected encounter rate when $d_{\mathrm{ij}}=0$, indicating direct overlap of an activity center with a trap, and σ is the scale parameter of the half‐normal detection function. The expected encounter rate can covary as a function of trap‐ and individual‐specific attributes, or by trapping occasion for sampling efforts with multiple occasions (e.g., $\lambda_{\mathrm{ijk}}$ for K>1).

We assume the encounter histories for the N observed individuals arise following $y_{\mathrm{ijk}}^{\mathrm{true}}∼\mathrm{Poisson}\left(\lambda_{\mathrm{ijk}}\right)$, though other count distributions could also be used. The true encounter frequencies $y_{\mathrm{ijk}}^{\mathrm{true}}$ for the n individuals with at least one detection are what would be observed if all samples were individually identifiable. Under a Bayesian approach to capture–recapture with unknown N, data augmentation can be used to estimate the number of unobserved individuals (Royle et al., 2007). We augment the n observed encounter histories with *M* − *n* “all‐ zero” histories, choosing a value such that M≫N. The likelihood for the zero‐inflated true encounter frequencies $y_{\mathrm{ijk}}^{\mathrm{true}}$ is then modified by a partially latent binary indicator variable $z_{i}$ that describes the membership of individual *i* in the population:

$$y_{\mathrm{ijk}}^{\mathrm{true}}|z_{i}∼\mathrm{Poisson}\left(\lambda_{\mathrm{ijk}}∙z_{i}\right)$$

Under this specification, $Pr\left(z_{i}=1\right)=1$ for the *n* observed individuals, and $z_{i}∼\mathrm{Bernoulli}\left(\psi \right)$ for the entire collection of M individuals. Population size can then be derived from the sum of indicators, $N=\sum z_{i}$ (realized *N*) or from the product M∙ψ (expected *N*), and density can be derived from dividing the population size by the area of the state space, D=N/‖S‖. Many other SCR observation models and specifications are possible depending on sampling design (Royle et al., 2014).

**Figure 1:**
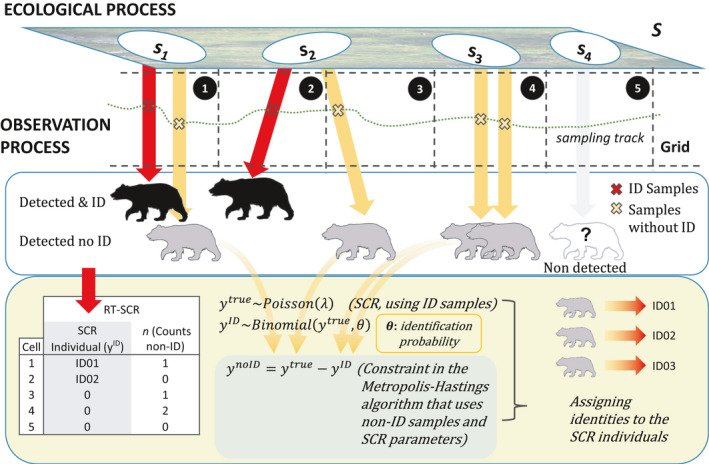
Graphical depiction of the random thinning spatial capture–recapture model. Random thinning SCR is hierarchical model with two processes: ecological (population size and location—$s_{i}$—of individuals) and observation. In this model (like in standard SCR), the detection rate of each individual depends on (i) Euclidean distance between individual's locations and traps (centroids of polygonal grid in the study case); (ii) baseline detection rate ($\lambda_{0}$) that here depends on sampling effort (length of transect in each polygon); and (iii) the scale parameter (σ) from the half‐normal detection function, that describes the animal movement. In the observation process, we obtain two types of data: encounters with identification ($y^{\mathrm{ID}}$) and non‐ID data ($y^{\mathrm{noID}}$) or counts. Random thinning SCR model uses ID data (in red) like in standard SCR to make inferences about population size and individuals' distribution (including nonobserved individuals, in gray), but also uses the counts (in orange) with a constraint ($y^{\mathrm{noID}}=y^{\mathrm{true}}‐y^{\mathrm{ID}}$) using a Metropolis–Hastings algorithm—in a mechanistic approach—to make a probabilistic reconstruction of the true encounter frequencies ($y^{\mathrm{true}}$), thus assigning identities to non‐ID samples

The process of assigning individual identities to samples in capture–recapture can be conceptualized as a random thinning process, where samples lose their individual identities at random with a probability 1‐θ. This process produces two types of data sets, one with individual identities and one without. Hereafter, this approach will be called the “random thinning SCR model” (see Figure 1 and DAG in Appendix S1). Thus, the new feature of our model is a submodel for individual identification conditional on the true encounter frequencies $y_{\mathrm{ijk}}^{\mathrm{true}}$. We define $y_{\mathrm{ijk}}^{\mathrm{ID}}$ to be identified samples from individual *i,* at trap *j* on occasion *k*. Then, we assume:

$$y_{\mathrm{ijk}}^{\mathrm{ID}}∼\mathrm{Binomial}\left(y_{\mathrm{ijk}}^{\mathrm{true}},\theta \right)$$

The individual identities of unrecognizable encounter frequencies $y_{\mathrm{ijk}}^{\mathrm{noID}}$ are then latent and $y_{\mathrm{ijk}}^{\mathrm{noID}}=y_{\mathrm{ijk}}^{\mathrm{true}}‐y_{\mathrm{ijk}}^{\mathrm{ID}}$. For the unidentified samples, we only observe the trap by occasion counts summed across captured individuals, ${\mathrm{nnid}}_{\mathrm{jk}}=\sum_{i=1}^{N}y_{\mathrm{ijk}}^{\mathrm{noID}}$. Thus, the same individual could be in both encounter histories —identified and not—at the same trap on the same sampling occasion. Note also that individuals with unidentified samples are not required to also be in the set of identified samples.

We fit this model in NIMBLE (NIMBLE Development Team, 2019) using a custom Metropolis–Hastings update for $y_{\mathrm{ijk}}^{\mathrm{true}}$ that obeys the constraint $y_{\mathrm{ijk}}^{\mathrm{noID}}=y_{\mathrm{ijk}}^{\mathrm{true}}‐y_{\mathrm{ijk}}^{\mathrm{ID}}$. This Metropolis–Hastings sampler was used because the full conditional distribution for $y_{.\mathrm{jk}}^{\mathrm{true}}$ used by Chandler & Royle (2013) is no longer valid when some individual identities may be known for the same individuals that also have latent identity samples (Appendix S2) and there is no default function implemented in NIMBLE to do this. We provide 2 versions of this sampler with and without the K dimension, which is faster when there are not occasion‐specific covariates or behavioral responses to capture (Appendix S2).

### 2.2 Simulation

We simulated 12 scenarios with 100 data sets in each scenario, in densities close to previous SCR density estimates from several species. We explored each case to assess the accuracy and precision of density estimates for the random thinning SCR model compared to a standard SCR model. We used two different trapping arrays, with higher density scenarios simulated on the smaller array requiring a lower population size (N) to achieve the desired density (D), thus reducing computation time for the simulation study. We explored low population densities (individuals/unit^2^) equivalent to population size (0.1 individuals/km^2^) found for Iberian lynx *Lynx pardinus* (Jiménez, Nuñez‐Arjona, et al., 2019) with a trap array 12 × 12 J=144and unit spacing with a baseline encounter rate $\lambda_{0}=0.5$. We also used two scenarios with a smaller trapping array (6×6;J=36) and unit spacing, with two levels of population size ($N\in \left\{20,50\right\};D\in \left\{0.35,0.89\right\}$ individuals/unit^2^), which can be viewed as density from stone marten *Martes foina* and fox *Vulpes vulpes* populations, respectively (Jiménez, Nuñez‐Arjona, et al., 2019). We generated encounter data with K=10surveys, with a baseline encounter rate $\lambda_{0}=0.65$and half‐normal scale parameter σ=0.5units (large array), and the same Kand σ, and $\lambda_{0}=0.5$(small array) simulating populations from low to high density, corresponding to low to high levels of home range overlap across individuals. Across the three density scenarios, we used four levels of identification probability $\left(\theta \in \left\{0.10,0.20,0.30,0.40\right\}\right)$(Appendix S3).

For the simulated data sets, we calculated the number of individuals captured, number of captures with identification ("ID"), captures without identification ("non‐ID"), number of recaptures, and number of spatial recaptures (mean and 95% confidence interval, see Appendix S3). Both the random thinning and regular SCR models were fit using NIMBLE (NIMBLE Development Team, 2019) in R (R Core Team, 2020) (Appendix S2). We fitted the random thinning model using both identified and unidentified samples, and we fitted the standard SCR models to the subset of encounter histories of identified individuals. In each case, we ran 3 chains of 50,000 (standard SCR models)‐500,000 iterations (random thinning SCR models), discarding 5,000–50,000 iterations as burn‐in and thinning by 5 or 100, respectively. We compared the posterior mean, median, and mode for point estimates. We calculated the root‐mean‐square error (RMSE) and the relative bias (RB) using the package SimDesign (Chalmers, 2020) in R and compared the improvements in RMSE using random thinning SCR models. We also calculated the coverage rates for the 95% highest posterior density (HPD) intervals for population sizes.

### 2.3 Application

**Figure 2:**
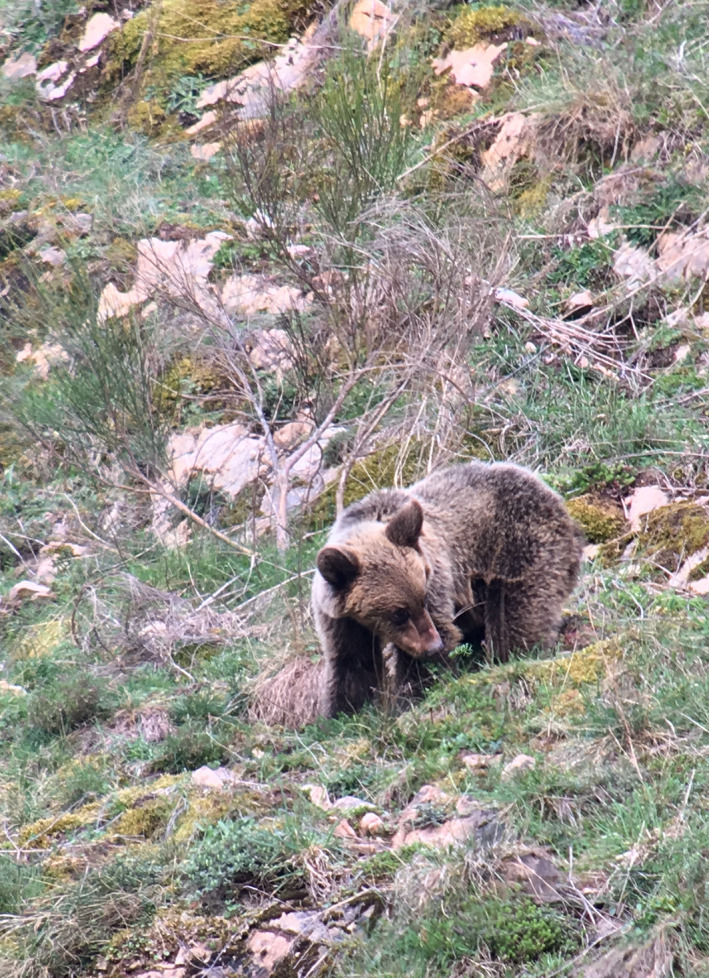
Brown bear (*Ursus arctos*) in Oriental Cantabrian Mountains (North Spain) in an “Escobal” (shrubland dominated by *Cytisus scoparius*). Credit: Jonathan Rodríguez‐Ramiro

The brown bear (Figure 2) is considered an “endangered species” under Spanish law (Real Decreto 139/2011, 2011) and is a “priority species” and a “species of community interest in need of strict protection” according to the European Community Habitat Directive (Council Directive 92/43/EEC, 1992). Brown bear in Spain form two separate nuclei: a small population (ca. 40 individuals) in the Pyrenees (Fundacion Oso Pardo, 2020), a mountain range between Spain and France, and a larger population in the Cantabrian Mountains (300 km along the northern coast of Spain; highest peak: 2,648 m). The Cantabrian population is fragmented across a mountain range into two subpopulations (Western and Eastern) separated by 50 km, and the total population was estimated at 200 individuals (95% CI: 183–278; Pérez et al., 2014) in 2014. We applied the random thinning SCR model to the smallest subpopulation, in the Eastern Cantabrian Mountains, using the data from Lopez‐Bao et al. (2020). Between November and December 2017, 128 bear fecal samples were collected, along with 23 hair samples. For each noninvasive sample, individual identification was attempted using both microsatellites and single nucleotide polymorphisms (SNPs). Lopez‐Bao et al. (2020) obtained 94 genotypes using SNPs (60% success; 68% (*n* = 87) for scats and 30% (*n* = 7) for hair). From those genotypes, they identified 33 different individuals. Lopez‐Bao et al. (2020) compared the two data sets (microsatellites versus. SNPs) and conducted population estimates using spatial capture–recapture models. They fit standard SCR models discretizing the space by a grid of hexagonal polygons and using the track length in each polygon as an effort covariate for the baseline detection rate $\lambda_{0}$:

$$\log\left(\lambda_{0}\left[j\right]\right)=\alpha_{0}+\alpha_{2}\times L\left[j\right]+\alpha_{3}\times {\left(L\left[j\right]\right)}^{2}$$

**Figure 3:**
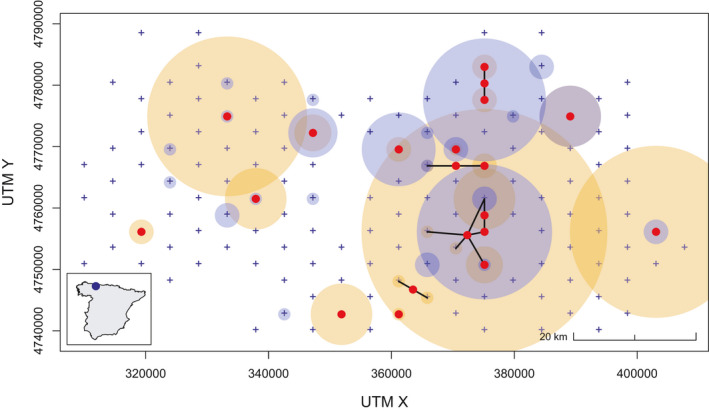
Locations of 144 cell centroids from the hexagonal grid (blue crosses) for sampling bears distributed across a 2,624 km^2^ Eastern Cantabrian Mountains region, in North Spain (inset map), and spiderplot (black lines: spatial recaptures; red points: average capture location from each individual). Encounters of known ID (light blue circles) and unknown ID (light gold circles) samples collected during September–December 2019 are illustrated. The size of the circles represents the number of captures (i.e., number of genotyped samples) at each cell

The full details and data of this study can be found in Lopez‐Bao et al. (2020). We used the SNP data and fit the random thinning SCR model. We also fit a standard SCR model using the known ID encounters without incorporating the unknown ID detections. The state space S was defined by a minimum convex polygon around the trap locations $\left(J=144\right)$ in a total area of 2,624 km^2^, buffered by 2.5×σ km (Figure 3). Each trap had K=1 survey. For both models, we used data augmentation with M=300 and calculated posterior summaries from 50,000 iterations generated by 3 chains of 55,000 iterations with a burn‐in of 5,000. We confirmed model convergence by examining trace plots and ensuring that the potential scale reduction factor ($\hat{R}$) statistic for each parameter was <1.1 (Gelman et al., 2013).

## 3 RESULTS

### 3.1 Simulations

Summary statistics of the posterior mean, median, and mode from our simulations are given in Appendix S4. The posterior mode is approximately unbiased as a point estimator at low density and ID rate. At medium density, the behavior of the posterior mode and median is similar, but at highest ID rate, we only found small differences between the posterior mean and median. In general, point estimates become more similar when the *N*/*D* posteriors are less right skewed, which occurs both when *D* and the percentage of identified samples increase. At high density, all point estimators are similar (Appendix S4). This is consistent with Chandler & Royle (2013), which noted that the skew in posterior distribution for *N* in unmarked SCR diminished as the number of marked individuals increased. Therefore, we used the posterior median as the point estimator.

**Figure 4:**
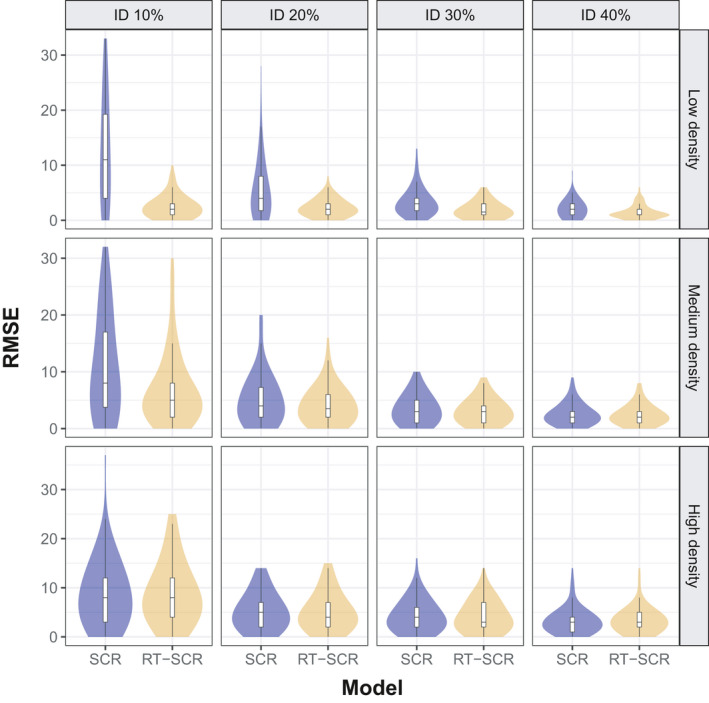
Violin plot of RMSE from population size estimates ($N\in \left\{20,20,50\right\}$ in three levels of density ($d\in \left\{0.1,0.4,0.9\right\}$ using standard SCR (light blue) and random thinning SCR (light gold) models for four levels of identification rate (θ∈{10%, 20%, 30%, 40%})

In the simulations, the improvement in population size estimates using the random thinning SCR model is more important in low‐density scenarios and is greater for lower rates of individual identification (Figure 4 and Appendix S4). For θ=0.1 (10% individual identification), there is a 79% RMSE reduction from 15.62 (SCR) to 3.28 (random thinning SCR), compared to a smaller (28.2%) reduction from 2.62 to 1.88 at θ=0.4. In medium‐density scenarios, there is a 32.2% RMSE reduction from 13.64 to 9.23 for θ=0.1, which decreases from 3.14 to 2.98 for θ=0.4 (5%). In high‐density situations, there is no improvement with the use of the random thinning SCR model. The improvement in the scale parameter estimate of the half‐normal detection function (σ) is similar: At low density, it is very substantial (from 0.19 to 0.04; 78.9%) at lower rates θ=0.1, but the improvement is reduced (0.05 to 0.03; 40%) at θ=0.4. At medium density, the RMSE improvement was from 0.19 to 0.07 (63.2%) at θ=0.1 and from 0.06 to 0.04 (33.3%) at θ=0.4. At high density, the RMSE improvement is less pronounced: from 0.08 to 0.05 (37.5%) at θ=0.1 and no improvement at θ=0.4. The baseline detection rate ($\lambda_{0}$) is the parameter with the greatest RMSE improvement (81.8%), from 0.44 to 0.08 (low density and θ=0.1) and from 0.40 to 0.15 (62.5%, high density and θ=0.4) (Appendix S4). In summary, RMSE reduction in the model's parameters by using random thinning SCR versus SCR is greater at lower levels of θ, and decreases as density increases (Appendix S3).

**Figure 5:**
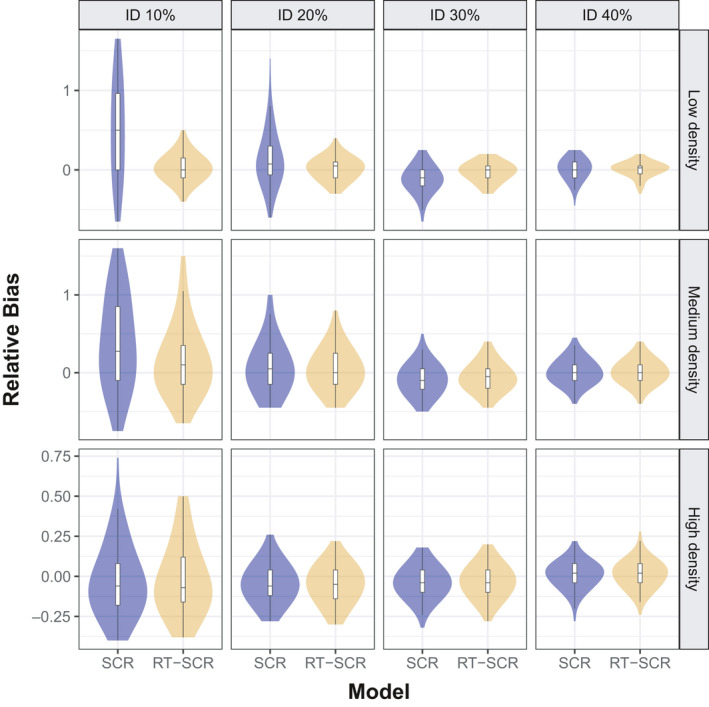
Violin plot of relative bias from population size estimates ($N\in \left\{20,20,50\right\}$ in three levels of density ($d\in \left\{0.1,0.4,0.9\right\}$ using standard SCR (light blue) and random thinning SCR (light gold) models for four levels of identification rate (θ∈ {10%, 20%, 30%, 40%})

Relative bias (RB) has the same behavior as RMSE. For θ=0.1, there is a 92.4% RB reduction from 0.66 (SCR) to 0.05 (random thinning SCR), and from 0.03 to 0.02 (3.3%) at θ=0.4. In medium‐density situations, there is a RB reduction from 0.54 to 0.26 (51.9%) for θ=0.1, which is almost the same values (0.03) in θ=0.4. In high‐density scenarios, the improvement in relative bias is minimal (Figure 5). Sigma was less biased using the random thinning SCR model at low and medium density, but there was no improvement at high density (Figure 5 and Appendix S4). Coverage of 95% HPD intervals was close to the nominal values in all cases (Appendix S4).

In scenarios with low density (*D* = 0.1, *N* = 20) and θ=0.1, the simulated data sets resulted in no spatial recaptures in the SCR data with individual identities for 33/100 data sets. In these cases, the random thinning SCR model was unbiased and worked correctly in situations where using an SCR model would be inadvisable. In such cases, the full data sets (prethinning) included spatial recaptures, which were lost in the ID random thinning, process, but remained in our data as unidentified capture events. These latent identity observations were used in the random thinning SCR model to probabilistically reconstruct latent spatial recapture events, allowing parameters to be estimated with minimal bias (RMSE = 3.83 and RB = −0.01), whereas the spatial scale parameter was estimated with strong bias in the reduced data. Similarly, for simulated data sets with only one spatial recapture (36 cases) using standard SCR, RMSE = 11.61 and RB = 0.31. Using random thinning SCR, RMSE = 3.06 and RB = 0.01, indicating a large improvement (73.6% and 96.8%, respectively). For two or more spatial recaptures (31 cases) using standard SCR, RMSE = 6.93 and RB = 0.08, and using the random thinning SCR model, RMSE = 2.34 and RB = 0.02 (an improvement of 66.2% and 75%, respectively).

According to our simulations, the random thinning SCR model performed well when density was low. For example, using a 12 × 12 grid of detectors and *N* = 20, even when the range of individuals detected was 4–10, 4–14 individuals were identified, 0–5 individuals were recaptures (0–4 spatial) and 87–143 detections could not be identified, and the population size error was quite low (RMSE = 3.5) ([Link], [Link]).

In summary, our simulations showed the random thinning SCR model yielded higher posterior precision than the standard SCR model at low density. At medium density, the improvement was moderate, and at high density, there was no improvement over standard SCR models. In all cases, the improvement increased as the individual identification rate (θ) decreased.

### 3.2 Brown bear application

In applying the model to the brown bear data set, we used the posterior median as the point estimate (appropriate at low density and high identification rate—see Appendix S4). The estimate of bear density was slightly (0.8%) lower for the random thinning SCR model (1.019 ± 0.172 bear/100 km^2^) than for the standard SCR model (1.027 ± 0.195 bear/100 km^2^; Table 1). By incorporating the unknown ID encounters (see Figures 1 and 3) into the random thinning SCR model, the CV of the density posterior was reduced by 11.8%. The movement parameter (σ) was also smaller (13.6%) for the random thinning SCR model than for the standard SCR model, and the CV was reduced by 31.6%. The increased baseline encounter rate, $\lambda_{0}$, logically reflected the additional encounters used by the random thinning SCR model (Table 1).

**Table 1:** Posterior summary statistics for both a standard spatial capture–recapture (SCR) model and the random thinning SCR model for a bear population in a 2,624 km^2^ (and 6 km buffer) region in the Eastern Cantabrian Mountains in Spain

Standard SCR			BCI	
	Median	*SD*	2.50%	97.50%
$\hat{D}$	1.027	0.195	0.715	1.479
${\hat{\alpha }}_{0}$	−0.240	0.275	−0.806	0.277
${\hat{\alpha }}_{2}$	1.525	0.266	1.058	2.086
${\hat{\alpha }}_{3}$	−0.171	0.058	−0.294	−0.066
$\hat{\psi }$	0.419	0.083	0.278	0.606
$\hat{\sigma }$	0.236	0.019	0.202	0.280

**Random thinning SCR**			BCI	
	Median	*SD*	2.50%	97.50%
$\hat{D}$	1.019	0.172	0.740	1.405
${\hat{\alpha }}_{0}$	0.915	0.190	0.542	1.281
${\hat{\alpha }}_{2}$	1.006	0.203	0.627	1.427
${\hat{\alpha }}_{3}$	−0.094	0.048	−0.195	−0.003
$\hat{\theta }$	0.599	0.039	0.520	0.675
$\hat{\psi }$	0.416	0.075	0.288	0.581
$\hat{\sigma }$	0.204	0.013	0.181	0.231

Note

We used the posterior median for all parameters, and presented the standard deviation and 95% Bayesian credible interval (BCI). $\hat{D}$ is population density (individuals/100 km^2^); ${\hat{\alpha }}_{0}$ is the intercept for effort; ${\hat{\alpha }}_{2}$ is the slope for effort; ${\hat{\alpha }}_{3}$ is the quadratic parameter for effort; $\hat{\theta }$ is the identification rate; $\hat{\psi }$ is the parameter for data augmentation; and $\hat{\sigma }$ is the scale parameter for the half‐normal distribution, related to movement of animals.

## 4 DISCUSSION

We demonstrated that the integration of unknown ID encounters with known ID encounters in an SCR modeling framework can improve the precision of density estimates while making use of available data that are often discarded in studies using noninvasive sampling techniques. A key aspect of the random thinning model that we proposed is that there is a natural mechanistic dependence between the unknown ID encounters and the known ID encounters in that they can arise from the same visitation of an individual to a location. This model structure allows for other count distributions (e.g., negative binomial) to be substituted for the Poisson encounter model without introducing dependence between sample types, and allows for variable thinning rates (discussed below).

Wildlife surveys that use remote camera trapping or passive/active collection of genetic material commonly produce encounter data that can be attributed to species at a much higher rate than to individuals. Approaches to population size estimation using capture–recapture, including the SCR model extensions (Royle et al., 2014), have traditionally required certainty in the assignment of identity to encounter data. This limitation can result in discarded information, which in other forms might be useful for modeling species distributions and/or habitat associations (e.g., Long et al. 2011). We illustrated that the inclusion of species detections (i.e., unknown ID encounters) provided useful gains in precision even under a basic model structure; further model complexity involving relationships between spatial environmental covariates and density or encounter probability (e.g., Royle et al.2013; Sutherland et al. 2015) would be expected to benefit similarly from the additional information that uses random thinning SCR.

In the brown bear application, density was very low but the proportion of identified individuals θ was high enough (~0.60) that a standard SCR model using only known ID encounters was able to provide a reasonable estimate of density, but even in these cases and by the configuration of detectors and the sigma value, we found an small improvement of the precision. Chandler & Royle (2013) demonstrated a rapid improvement in the RMSE of the posterior density estimates of their spatial model as the number of marked individuals in the sample increased from zero. Similarly, Sollmann et al. (2013) were able to estimate density with incomplete individual identification using a SMR model even with a small number of marked individuals. In both of these applications, the marking status of each encounter was known with certainty. For the Chandler & Royle (2013) model applied to data without marked individuals, the spatial information associated with encounters was only useful when the study design ensured spatial correlation in the unidentified counts. Our result suggests that in low‐density populations, unidentified samples can be very informative about parameters, and this information results in improved estimates of all model parameters. Sollmann et al. (2013) used telemetry data to directly inform the model about individual movement and suggested density estimation would have otherwise been impossible. In the random thinning SCR model, telemetry data could be incorporated to improve this parameter estimate, particularly if there are few spatial recaptures.

The random thinning SCR model can be seen as an intermediate model between unmarked SCR (θ=0; Chandler & Royle, 2013) and standard SCR (θ=1). Unlike the standard SMR model, the random thinning SCR model described here does not separate individuals into “marked” and “unmarked” classes. Note, this is the same “random thinning” model previously used by Jiménez, Chandler, et al. (2019) where it was applied to only a subset of individuals that were marked in an SMR framework, whereas we apply it to all individuals. We consider that all individuals may produce identified and unidentified samples, and the process of individual identification occurs with a success rate θ such that observed data with individual identities are a thinned version of the true encounter histories. The success rate can be a function of the random failure to map an encounter to an individual (e.g., poor quality photograph or DNA) or a deterministic decision based on study design considerations (e.g., Chandler & Clark, 2014).

Variation in the thinning rate θ could arise in a variety of ways. Individual, trap, and occasion‐specific covariates or random effects can be modeled on θ. For example, individuals might vary in their identifiability in camera trap photographs, or DNA amplification might be a function of occasion or trap by occasion weather covariates. In fact, our random thinning model could potentially be used for studies where only a subset of naturally marked individuals are identifiable, which are currently analyzed using SMR. This approach would obviate the need to classify photographs as belonging to “marked” or “unmarked” individuals, which is often difficult to determine using natural marks in photographs, but at the expense of reduced information in the unidentified observations (mark status). For this SMR‐type case, we believe a finite mixture on θ could be used, with one mixture component for θ fixed to 0 to accommodate truly unidentifiable individuals, and θ for the identifiable component of the population and the mixing proportion to be estimated. We are unsure if such a model would produce competitive estimates with SMR, even if it removes the possibility of errors in classifying the mark status of samples, but recommend it be studied further. Sample‐level covariates and random effects could also be used (e.g., to accommodate variable DNA sample quality/quantity) but those require the code be modified to update the individual identity of one sample at a time.

An alternative model for combining identified and unidentified DNA samples is that of (Augustine et al., 2020), where each genetic locus is regarded as a partial identity covariate, potentially observed with error. That model has two advantages over the model we consider here. First, it allows for the possibility that there may be errors in the individual identities assigned to the known identity samples. These errors should generally be rare, but they cannot be removed with certainty. Second, the Augustine et al. (2020) model can utilize the partial genotype scores associated with the unidentified samples to extract more information out of these samples.

Another model, the multiple observation process (MOP) model for combining SCR data and detections of unidentified individuals, was recently proposed by Tourani, Dupont, Nawaz, & Bischof (2020). Rather than conditioning on the latent encounter histories as we have done, the MOP model treats the two data sets as independent, except for sharing the same expected values for the detection process, modified by the thinning rate; however, in general, these data sets will not be independent because the detection methods are colocated (Clare et al. 2017). These two data types can be regarded as independent in a special case where i) the true encounters are Poisson‐distributed, and ii) the thinning rate is fixed for all samples (following standard results for thinning Poisson processes; Chiu et al., 2013, p. 161). If this holds, the two sets of Poisson counts (ID and no‐ID) can be converted to the Bernoulli data used by MOP (see Appendix S5). This Poisson requirement for independence between the ID and no‐ID samples prevents the MOP model from considering that the true encounters came from other count distributions, for example, the negative binomial. Additionally, we can expect a loss of precision using detections instead of counts. In random thinning SCR model, because it probabilistically reconstructs the capture history samples, the dependence issue pointed out by Clare, McKinney, DePue, and Loftin (2017) it is not a problem. Another difference of our random thinning SCR model is that by conditioning on the latent encounter history, the model can accommodate a behavioral response to capture.

Computation is slow for the random thinning SCR model, and even using the faster version in NIMBLE without temporal variation on $\lambda_{0}$ or θ, is slower to converge than the standard SCR model (in which we will need 50,000 iterations with 3 chains, that requires 20–30 min using a 2.5 Ghz computer). Random thinning SCR requires a high number of iterations, especially at low rates of identified events (typically we will need 500.000–1,000,000 iterations with a thinning of 100 for a θ=0.1, which requires 3–6 hr). The mixing in Nimble can be improved and runtime reduced by changing the default update chosen by Nimble for the activity centers (Appendix S2).

According to our simulations ([Link], [Link]), the random thinning SCR model outperformed standard SCR model when density and θ were low. In high‐density scenarios with high identification rates, there is almost no improvement in precision from using the random thinning SCR model, and it is advisable to discard the unidentified samples and fit standard SCR models to the observed encounter histories (though these samples may still be useful in a modified version of the model that probabilistically reconstructs a latent behavioral response to capture). At intermediate values of density and θ, users would need to do simulations themselves that are in line with their specific applications to know the possible precision improvement. The probabilistic reconstruction of capture histories using the random thinning SCR is much more efficient in situations where there is little assignment uncertainty associated with unknown identities. The benefit of modeling the unidentified samples is greatest in scenarios where individual home range overlap is low, with sparse identified samples and abundant unidentified samples. Low‐ and medium‐density scenarios with rates of individual identification under 0.5 are common (e.g., Aziz et al., 2017; Hooker et al., 2015; Kendall et al., 2016; Molina et al., 2017; Murphy et al., ,^2016^, ^2018^; Ngoprasert et al., 2012; Sun et al., 2017), and the random thinning model may therefore be widely applicable.

## CONFLICT OF INTEREST

None declared.

## AUTHOR CONTRIBUTIONS

**Jose Jimenez:** Conceptualization (equal); formal analysis (lead); investigation (lead); methodology (equal); software (equal); supervision (lead); validation (lead); visualization (lead); writing–original draft (equal). **Be Augustine:** Conceptualization (equal); methodology (equal); software (equal); writing–original draft (equal). **Daniel W. Linden:** Conceptualization (equal); methodology (equal); software (equal); writing–original draft (equal). **Richard Chandler:** Conceptualization (equal); methodology (equal); software (equal); writing–original draft (equal). **J. Andrew Royle:** Conceptualization (equal); methodology (equal); software (equal); writing–original draft (equal).

## Supporting information

Appendix S1

Appendix S2

Appendix S3

Appendix S4

Appendix S5

## Data Availability

R scripts to simulate data and perform the analyses are available in online supporting information.
