# Supplementary material for: Spatial capture–recapture with random thinning for unidentified encounters
Source: Ecol Evol. 2020 Dec 8;11(3):1187–98. doi: 10.1002/ece3.7091 (PMC7863675; doi:10.1002/ece3.7091)
Supplement: Supplementary file 1 — Appendix S1 [file ECE3-11-1187-s001.docx]

Appendix 1. DAG


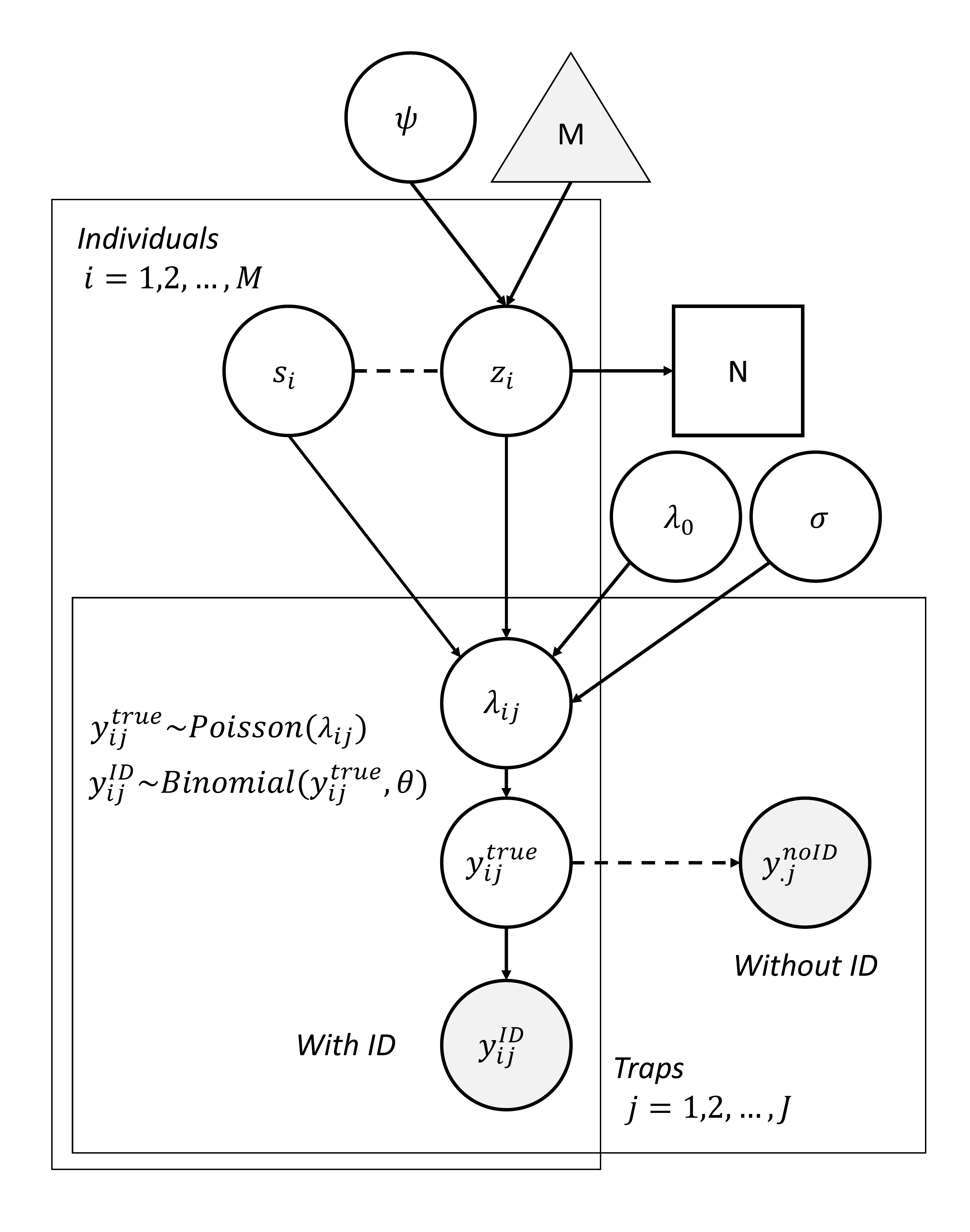


**Figure 1.** Directed acyclic graph of the random thinning-SCR model. Dashed lines represent objects linked along the first dimension and dashed arrows represent disaggregation of data from the individual level to the sample level. Grey nodes represent observed quantities (data) and white nodes unknown quantities (latent variables and model parameters). $y_{ij}^{true}$: true encounter frequencies (latent); $y_{ij}^{ID}$: identified encounters; $y_{.j}^{noID}$: non-ID encounters (counts); $\lambda_{ij}$: detection rate; $\lambda_{0}$: basal detection rate; $\theta$: thinning rate (probability of identification); $\sigma$: scale parameter from the half-normal detection function, that describe the animal movement; $s_{i}$: individuals activity centers; $z_{i}$: latent binary indicator variable that describes the membership of individual $i$ in the population; $\psi$: data augmentation parameter; $M$: data augmented; $N$: population size.
