# Supplementary material for: Spatial capture–recapture with random thinning for unidentified encounters
Source: Ecol Evol. 2020 Dec 8;11(3):1187–98. doi: 10.1002/ece3.7091 (PMC7863675; doi:10.1002/ece3.7091)
Supplement: Supplementary file 2 — Appendix S2 [file ECE3-11-1187-s002.docx]

Appendix 2. Random Thinning-SCR model and data simulator

1. Random-Thinning SCR code

## Nimble code

library(nimble)

## nimble version 0.10.0 is loaded.
## For more information on NIMBLE and a User Manual,
## please visit http://R-nimble.org.

##
## Attaching package: 'nimble'

## The following object is masked from 'package:stats':
##

####NIMBLE model
 NimModel <- nimbleCode({

 lam0 ~ dunif(0,5)
 sig ~ dunif(0,5)
 sig2 <- 2*sig^2
 psi ~ dbeta(1,1)
 id.prob ~ dunif(0,1) # Identification probability

 for(i in 1:M) {
 s[i,1] ~ dunif(xlim[1], xlim[2])
 s[i,2] ~ dunif(ylim[1], ylim[2])
 z[i] ~ dbern(psi)
 d2[i,1:J] <- (s[i,1]-x[1:J,1])^2 + (s[i,2]-x[1:J,2])^2
 lam[i,1:J] <- lam0*exp(-d2[i,1:J]/sig2)*z[i]

 for(j in 1:J) {
 for(k in 1:K){
 y.true[i,j,k] ~ dpois(lam[i,j]) # Model for complete capture histories
 y.ID[i,j,k] ~ dbin(id.prob, y.true[i,j,k]) # Model for ID capture histories
 }
 }
 }

 # nnid is used in IDSampler (Metropolis-Hasting sampler)

 N <- sum(z[1:M])
 D <- N/A
 })

## Metropolis-Hastings sampler

## sampler to jointly update y.noID[1:M,j,k] so that they sum to nnid[j,k]

*##* *We use the Chandler & Royle (2013) full conditional distribution for* $y_{.jk}^{true}$ *as the*

*## Metropolis-Hastings proposal distribution.*

IDSampler <- nimbleFunction(
 contains = sampler_BASE,
 setup = function(model, mvSaved, target, control) {
 # Defined stuff
 nnid<-control$nnid
 j<-control$j
 k<-control$k
 M<-control$M
 calcNodes <- model$getDependencies(target)
 },

 run = function() {
 lam.curr <- model$lam[1:M,j] #individual by trap expected counts

 switch.probs <- lam.curr[1:M]/sum(lam.curr[1:M])

 #propose new ID's for nnid[j,k]
 y.latent.curr <- model$y.true[1:M,j,k] - model$y.ID[1:M,j,k]
 y.latent.prop <- rmulti(1, nnid, switch.probs[1:M])
 model$y.true[1:M,j,k] <<- model$y.ID[1:M,j,k] + y.latent.prop

 # initial model logProb
 model_lp_initial <- model$getLogProb(calcNodes)

 # proposal model logProb
 model_lp_proposed <- model$calculate(calcNodes)

 # log-Metropolis-Hastings ratio
 log_MH_ratio <-(model_lp_proposed+ dmulti(y.latent.curr, nnid, switch.probs, log=TRUE))-
 (model_lp_initial + dmulti(y.latent.prop, nnid, switch.probs, log=TRUE))

 # Metropolis-Hastings step
 accept <- decide(log_MH_ratio)
 if(accept) {
 copy(from = model, to = mvSaved, row = 1, nodes = calcNodes, logProb = TRUE)
 } else {
 copy(from = mvSaved, to = model, row = 1, nodes = calcNodes, logProb = TRUE)
 }
 },
 methods = list( reset = function () {} )
)

1. Random-Thinning SCR code summarized over time

## Nimble code

library(nimble)

## nimble version 0.10.0 is loaded.
## For more information on NIMBLE and a User Manual,
## please visit http://R-nimble.org.

##
## Attaching package: 'nimble'

## The following object is masked from 'package:stats':
##

NimModel <- nimbleCode({

 lam0 ~ dunif(0,5)
 sig ~ dunif(0,5)
 sig2 <- 2*sig^2
 psi ~ dbeta(1,1)
 id.prob ~ dunif(0,1)

 for(i in 1:M) {
 s[i,1] ~ dunif(xlim[1], xlim[2])
 s[i,2] ~ dunif(ylim[1], ylim[2])
 z[i] ~ dbern(psi)
 d2[i,1:J] <- (s[i,1]-x[1:J,1])^2 + (s[i,2]-x[1:J,2])^2
 lam[i,1:J] <- lam0*exp(-d2[i,1:J]/sig2)*z[i]

 for(j in 1:J) {
 y.true[i,j] ~ dpois(lam[i,j]*K) # Model for complete capture histories
 y.obs[i,j] ~ dbin(id.prob, y.true[i,j]) # Model for ID capture histories
 }
 }

 # nnid is used in IDSampler (Metropolis-Hasting sampler)

 N <- sum(z[1:M])
 D <- N/A
 })

## Metropolis-Hastings sampler

## sampler to jointly update y.noID[1:M,j] so that they sum to nnid[j]

## We use the Chandler & Royle (2013) full conditional distribution for $y_{.jk}^{\mathrm{true}}$ as the

## Metropolis-Hastings proposal distribution.
 IDSampler <- nimbleFunction(
 contains = sampler_BASE,
 setup = function(model, mvSaved, target, control) {
 # Defined stuff
 nnidd<-control$nnidd
 j<-control$j
 M<-control$M
 calcNodes <- model$getDependencies(target)
 },

 run = function() {
 lam.curr <- model$lam[1:M,j] #individual by trap expected counts

 switch.probs <- lam.curr[1:M]/sum(lam.curr[1:M])

 #propose new ID's for nnid[j]
 y.latent.curr <- model$y.true[1:M,j]- model$y.ID[1:M,j]
 y.latent.prop <- rmulti(1, nnidd, switch.probs[1:M])
 model$y.true[1:M,j] <<- model$y.ID[1:M,j] + y.latent.prop

 # initial model logProb
 model_lp_initial <- model$getLogProb(calcNodes)

 # proposal model logProb
 model_lp_proposed <- model$calculate(calcNodes)

 # log-Metropolis-Hastings ratio
 log_MH_ratio<-(model_lp_proposed + dmulti(y.latent.curr, nnidd, switch.probs, log=TRUE)) -
 (model_lp_initial + dmulti(y.latent.prop, nnidd, switch.probs, log=TRUE))

 # Metropolis-Hastings step
 accept <- decide(log_MH_ratio)
 if(accept) {
 copy(from = model, to = mvSaved, row = 1, nodes = calcNodes, logProb = TRUE)
 } else {
 copy(from = mvSaved, to = model, row = 1, nodes = calcNodes, logProb = TRUE)
 }
 },
 methods = list( reset = function () {} )
 )

1. Data simulator and R+Nimble code (summarized over *k*)

### Define working directory

setwd('C:/...')

library(nimble)

## nimble version 0.10.0 is loaded.
## For more information on NIMBLE and a User Manual,
## please visit http://R-nimble.org.

##
## Attaching package: 'nimble'

## The following object is masked from 'package:stats':
##
## simulate

**library**(coda)
**library**(lattice)
**library**(scrbook)
**library**(MCMCvis)
**source**("SpiderPlotFunction.R")

### Funtions to use

e2dist <- function (x, y) { # Function from scrbook package to calculate the distance between

# locations in 2 matrices.
 i <- sort(rep(1:nrow(y), nrow(x)))
 dvec <- sqrt((x[, 1] - y[i, 1])^2 + (x[, 2] - y[i, 2])^2)
 matrix(dvec, nrow = nrow(x), ncol = nrow(y), byrow = F)
}

### Data simulator

SimSCR0<-function (N = 100, K = 5, lam0 = 0.35, sigma = 0.5, discard0 = TRUE,
 tel=2, n.locs=50, rnd = 2013) {
 set.seed(rnd)
 traplocs <- cbind(sort(rep(1:12, 12)), rep(1:12, 12))
 Dmat <- e2dist(traplocs, traplocs)
 ntraps <- nrow(traplocs)
 buffer <- 1.25
 Xl <- min(traplocs[, 1] - buffer)
 Xu <- max(traplocs[, 1] + buffer)
 Yl <- min(traplocs[, 2] - buffer)
 Yu <- max(traplocs[, 2] + buffer)
 sx <- runif(N, Xl, Xu)
 sy <- runif(N, Yl, Yu)
 S <- cbind(sx, sy)
 D <- e2dist(S, traplocs)
 lam <- lam0 * exp(-(D * D)/(2 * sigma * sigma))
 plot(traplocs, xlim=c(Xl,Xu), ylim=c(Yl,Yu), pch="+")
 points(S, col="blue", pch=16)
 Y <- array(NA, dim = c(N, ntraps, K))
 for (i in 1:nrow(Y)) {
 for (j in 1:ntraps) {
 Y[i, j, 1:K] <- rpois(K, lam[i, j])
 }
 }
 if (discard0) {
 Y2d <- apply(Y, c(1, 2), sum)
 ncaps <- apply(Y2d, 1, sum)
 Y <- Y[ncaps > 0, , ]
 }

 if (tel > 0) {
 itel <- sort(sample(1:tel, tel, replace = F))
 locs <- list()
 for (i in 1:tel) {
 lx <- rnorm(n.locs, S[itel[i], 1], sigma)
 ly <- rnorm(n.locs, S[itel[i], 2], sigma)
 locs[[i]] <- cbind(lx, ly)
 }
 }
 else {
 locs <- NULL
 itel <- NULL
 }

 list(Y = Y, traplocs = traplocs, xlim = c(Xl, Xu), ylim = c(Yl, Yu), N = N,
 lam0 = lam0, sigma = sigma, K = K, tel = tel, locs=locs, n.locs=n.locs, S=S)
}

## Data simulation

data <- SimSCR0(N=20, lam0=0.5, K=10, discard0=TRUE, sigma=0.5, tel=2, n.locs=50, rnd=1)


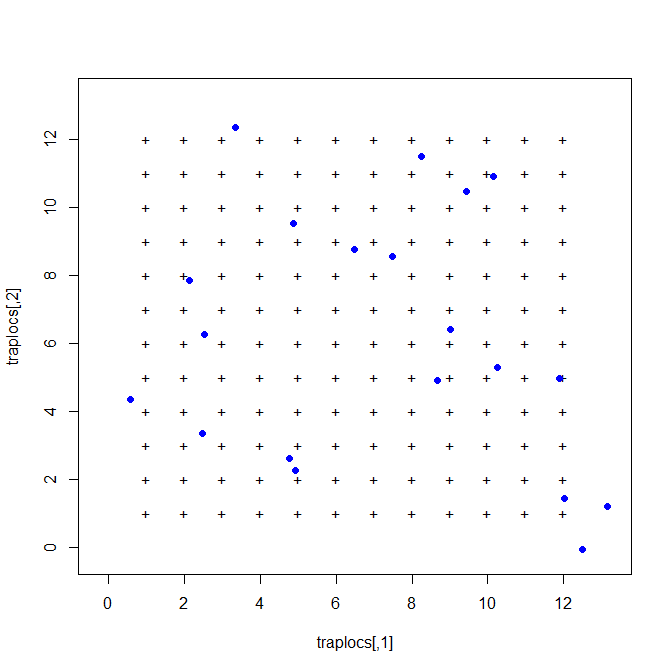


Figure 1. Data simulation. 12 x 12 detector grid, with 20 individuals (blue points), movement parameter σ=0.5 units, baseline detection rate $\lambda_{0}=0.65$, in a state space ($\left\| S \right\|=182.25$ units square).

Setting data

M <- 150
# Activity centres
S<-data$S
y<- data$Y # All capture histories

# Traps
X<- data$traplocs
X<-matrix(X, ncol=2)
J<-nrow(X) # Number of traps
K<-data$K # Number of sampling occasions

nind <- nrow(apply(y, c(1,2), sum)) # Number of individuals detected

set.seed(1960)
## Simulation of of ID individuals
id.prob <- 0.1 # ID rate
y.id1<-array(0,c(nind, J, K))
for(i in 1:nind){
 for(j in 1:J){
 for(k in 1:K){
 y.id1[i,j,k]<-rbinom(prob=id.prob, 1, y[i,j,k])
 }
 }
}

sum(y.id1) # ID events

## [1] 10

y.id<-y.id1[apply(y.id1,1,sum)>0,,] # Dropping non-ID histories

(nind2<-nrow(apply(y.id, c(1,3), sum))) # Number of ID individuals

## [1] 8

## [1] 8
M <- 80 # Data augmentation
yaug<-array(0,c(M,J,K))
yaug[1:nind2,,]<-y.id
sum(yaug) # Number of ID events

## [1] 10

# Non-ID capture frequencies
nnid<-apply(y,c(2,3),sum)-apply(y.id, c(2,3), sum)
sum(nnid) # Number of non-ID events

## [1] 122

nnidd<-apply(nnid,1,sum)

# State space
xlims <- data$xlim
ylims <- data$ylim
A <- diff(xlims)*diff(ylims) # State space area

# Capture plot
plot(X, pch="+",cex=1, xlim=xlims, ylim=ylims, main="", type="n")
tot<-apply(y.id, 2,sum)
## ID individuals
symbols(X, circles=tot/5, inches=F, bg="#EEAD0E66", fg=NULL, add=T)
## Non-ID
nID<-as.numeric(apply(nnid,1,sum))
symbols(X, circles=nID/5, inches=F,bg="#0000FF3F", fg=NULL, add=T)
points(X, pch="+", cex=1)
points(data$S, col="blue", pch=16)
spiderplot.Over(y.id, X, buffer=0,lwd=2)

Figure 2. The size of the circles represents the total number of detections at each detector. ID captures (grey circles) and non-ID counts (golden circles). Black lines are the spatial recaptures, and red points are the average between captures of the same individuals.

## Nimble model

library(nimble)
####NIMBLE model
NimModel <- nimbleCode({

 lam0 ~ dunif(0,5)
 sig ~ dunif(0,5)
 sig2 <- 2*sig^2
 psi ~ dbeta(1,1)
 id.prob ~ dunif(0,1)

 for(i in 1:M) {
 s[i,1] ~ dunif(xlim[1], xlim[2])
 s[i,2] ~ dunif(ylim[1], ylim[2])
 z[i] ~ dbern(psi)
 d2[i,1:J] <- (s[i,1]-x[1:J,1])^2 + (s[i,2]-x[1:J,2])^2
 lam[i,1:J] <- lam0*exp(-d2[i,1:J]/sig2)*z[i]

 for(j in 1:J) {
 for(k in 1:K){
 y.true[i,j,k] ~ dpois(lam[i,j]) # Model for all capture histories
 y.obs[i,j,k] ~ dbin(id.prob, y.true[i,j,k]) # Model for ID individuals
 }
 }
 }

 N <- sum(z[1:M])
 D <- N/A
})

## Metropolis-Hastings sampler

## sampler to jointly update y.un[1:M,j] so that they sum to n[j]
IDSampler <- nimbleFunction(
 contains = sampler_BASE,
 setup = function(model, mvSaved, target, control) {
 # Defined stuff
 nnidd<-control$nnidd
 j<-control$j
 M<-control$M
 calcNodes <- model$getDependencies(target)
 },

 run = function() {
 lam.curr <- model$lam[1:M,j] #individual by trap expected counts

 #Sample y[1:M,j] by reassigning n[j] using full conditional
 switch.probs <- lam.curr[1:M]/sum(lam.curr[1:M])

 #propose new ID's for nnid[j,k]
 y.latent.curr <- model$y.full[1:M,j]- model$y.obs[1:M,j]
 y.latent.prop <- rmulti(1, nnidd, switch.probs[1:M])
 model$y.full[1:M,j] <<- model$y.obs[1:M,j] + y.latent.prop

 # initial model logProb
 model_lp_initial <- model$getLogProb(calcNodes)

 # proposal model logProb
 model_lp_proposed <- model$calculate(calcNodes)

 # log-Metropolis-Hastings ratio
 log_MH_ratio <- (model_lp_proposed + dmulti(y.latent.curr, nnidd, switch.probs, log=TRUE)) -
 (model_lp_initial + dmulti(y.latent.prop, nnidd, switch.probs, log=TRUE))

 # Metropolis-Hastings step
 accept <- decide(log_MH_ratio)
 if(accept) {
 copy(from = model, to = mvSaved, row = 1, nodes = calcNodes, logProb = TRUE)
 } else {
 copy(from = mvSaved, to = model, row = 1, nodes = calcNodes, logProb = TRUE)
 }
 },
 methods = list( reset = function () {} )

)

## Constants

## CONSTANTS
constants<-list(nnid=nnid, # non-id events
 J=J, # no. traps
 M=M, # data augmentation
 K=K, # no. occasions
 xlim=xlims, # state space limits
 ylim=ylims,
 A=A) # state space size

str(constants)

## List of 7
## $ nnid: num [1:144, 1:10] 0 0 0 0 0 0 0 0 0 0 ...
## $ J : int 144
## $ M : num 80
## $ K : num 10
## $ xlim: num [1:2] -0.25 13.25
## $ ylim: num [1:2] -0.25 13.25
## $ A : num 182

## Data

yred<-**apply**(yaug,**c**(1,2),sum)
data <- **list**(y.obs=yred, *# ID histories*
 x=X) *# traps coordinates*
**str**(data)

## List of 2
## $ y.obs: num [1:80, 1:144] 0 0 0 0 0 0 0 0 0 0 ...
## $ x : int [1:144, 1:2] 1 1 1 1 1 1 1 1 1 1 ...

## Inits

# Initial values in NIMBLE must be carefully tailored to ensure that all nodes,
# particularly for latent y.true, begin at reasonable initial values. Any error
# here could invalidate the results
ys<-apply(yaug,c(1,2),sum)
s.start <- cbind(runif(M, xlims[1], xlims[2]), runif(M, ylims[1], ylims[2]))
d <- e2dist(s.start[1:M,], X)
lam0s<- runif(1,0.1,0.5)
sigs <- runif(1,0.3,0.8)

lam <- lam0s * exp( -(d^2)/(2 * sigs^2))

yi <- array(0, c(M, J, K)) # resighting array
for (j in 1:J) {
 for (k in 1:K) {
 if (nnid[j, k] > 0) {
 probs <- lam[ ,j]
 probs <- probs / sum(probs)
 latent.id <- sample(1:M, nnid[j,k], prob = probs, replace = FALSE)
 yi[latent.id , j, k] <- 1
 }
 } # end of k
} # end of j

yis<-apply(yi,c(1,2),sum) + apply(yaug,c(1,2),sum)
zst<-apply(yis, 1, sum); zst[zst>0]<-1
id.prob.s<-sum(yaug)/(sum(yaug)+sum(nnid))

inits <- list(z=zst, # z inits
 s=s.start, # s inits
 lam0=lam0s, # baseline detection rate
 sig=sigs, # movement parameter
 id.prob=id.prob.s, # detection rate
 y.full=yis) # latent true histories
str(inits)

## List of 6
## $ z : num [1:80] 1 1 1 1 1 1 1 1 0 0 ...
## $ s : num [1:80, 1:2] 10.31 13.03 11.85 11.67 5.43 ...
## $ lam0 : num 0.281
## $ sig : num 0.462
## $ id.prob: num 0.0758
## $ y.full : num [1:80, 1:144] 0 0 0 0 0 0 0 0 0 0 ...

## Parameters

params <- c('psi', 'lam0', 'sig', 'N', 'D', 'id.prob')

##

## Running the model

# Compile and run using NIMBLE
start.time<-Sys.time()
Rmodel <- nimbleModel(code=NimModel,
 constants=constants,
 data=data,
 inits=inits,
 check=FALSE,
 calculate=FALSE)

## defining model...

## building model...

## setting data and initial values...

## checking model sizes and dimensions... This model is not fully initialized. This is not an error. To see which variables are not initialized, use model$initializeInfo(). For more information on model initialization, see help(modelInitialization).
## model building finished.

Cmodel <- compileNimble(Rmodel)

## compiling... this may take a minute. Use 'showCompilerOutput = TRUE' to see C++ compilation details.
## compilation finished.

conf <- configureMCMC(Rmodel,monitors=params, thin=50, useConjugacy = TRUE,
 onlySlice=TRUE)

## ===== Monitors =====
## thin = 50: psi, lam0, sig, N, D, id.prob
## ===== Samplers =====
## slice sampler (11764)
## - lam0
## - sig
## - psi
## - id.prob
## - s[] (160 elements)
## - z[] (80 elements)
## - y.full[] (11520 elements)

#conf$printSamplers()

#### --- load custom samplers at end of script --- ####
# replace with new sampler for y (sample without replacement with sum
# to n[j,k] constraint)
conf$removeSampler("y.full")
for(j in 1:J){
 conf$addSampler(target = paste(paste("y.full[1:",M,", ",j,"]"), sep=""),
 type = 'IDSampler', # If running in parallel, replace
 # type="IDSampler"
 # with type=IDSampler
 control = list(nnidd = nnidd[j], j=j, M=M),
 silent = TRUE)
}
# Rebuild and compile with new sampler
conf$removeSamplers("s")
ACnodes <- paste0("s[", 1:constants$M, ", 1:2]")
for(node in ACnodes) {
 conf$addSampler(target = node,
 type = "AF_slice",
 control = list(adaptScaleOnly = TRUE),
 silent = TRUE)
}


Rmcmc <- buildMCMC(conf)
Cmcmc <- compileNimble(Rmcmc, project = Rmodel)

## compiling... this may take a minute. Use 'showCompilerOutput = TRUE' to see C++ compilation details.
## compilation finished.

outNim <- runMCMC(Cmcmc, niter = 500000, nburnin = 50000, nchains = 3, inits=inits,
 setSeed = TRUE, progressBar = TRUE, samplesAsCodaMCMC = TRUE)

## runMCMC's handling of nburnin changed in nimble version 0.6-11. Previously, nburnin samples were discarded *post-thinning*. Now nburnin samples are discarded *pre-thinning*. The number of samples returned will be floor((niter-nburnin)/thin).
## running chain 1...

## |-------------|-------------|-------------|-------------|
## |-------------------------------------------------------|

## running chain 2...

## |-------------|-------------|-------------|-------------|
## |-------------------------------------------------------|

## running chain 3...

## |-------------|-------------|-------------|-------------|
## |-------------------------------------------------------|

end.time<-Sys.time()
end.time-start.time

## Time difference of 15.97694 hours

## Summarize posteriors and inspecting the convergence

summary(outNim)

##
## Iterations = 1:9000
## Thinning interval = 1
## Number of chains = 3
## Sample size per chain = 9000
##
## 1. Empirical mean and standard deviation for each variable,
## plus standard error of the mean:
##
## Mean SD Naive SE Time-series SE
## D 0.12363 0.02559 0.0001557 0.0006331
## N 22.53093 4.66337 0.0283804 0.1153908
## id.prob 0.08228 0.02363 0.0001438 0.0001438
## lam0 0.35771 0.06714 0.0004086 0.0007960
## psi 0.28647 0.07526 0.0004580 0.0014370
## sig 0.57428 0.05515 0.0003356 0.0009539
##
## 2. Quantiles for each variable:
##
## 2.5% 25% 50% 75% 97.5%
## D 0.08779 0.10425 0.12071 0.13717 0.1811
## N 16.00000 19.00000 22.00000 25.00000 33.0000
## id.prob 0.04241 0.06526 0.08017 0.09695 0.1345
## lam0 0.23850 0.31012 0.35318 0.40055 0.5010
## psi 0.16039 0.23280 0.27938 0.33207 0.4525
## sig 0.47152 0.53605 0.57292 0.61064 0.6873

xyplot(outNim)

REFERENCES

Chandler, R. B., & Royle, J. A. (2013). Spatially-explicit models for inference about density in unmarked populations*. The Annals of Applied Statistics*, 7(2), 936–954. doi:10.1214/12-AOAS610
