# Supplementary material for: Spatial capture–recapture with random thinning for unidentified encounters
Source: Ecol Evol. 2020 Dec 8;11(3):1187–98. doi: 10.1002/ece3.7091 (PMC7863675; doi:10.1002/ece3.7091)
Supplement: Supplementary file 3 — Appendix S3 [file ECE3-11-1187-s003.docx]

Appendix 3: Simulations Specifications

Table 1. Three scenarios (A, B and C) simulated with 144, 36 and 36 traps. *Trap. Dist.*, distance between traps; *Size S*, state space size; *ID*, identification rate; *N*, population size simulated; $\sigma$, half-normal scale parameter, and $\lambda_{0}$, baseline detection rate. In each scenario we conducted 100 simulations.

| Scenario | nº traps | Traps dist. | Size S | ID | N | σ | $\lambda_{0}$ |
| --- | --- | --- | --- | --- | --- | --- | --- |
| A | 144 | 1 | 182.25 | 0.1 | 20 | 0.5 | 0.50 |
| A | 144 | 1 | 182.25 | 0.2 | 20 | 0.5 | 0.50 |
| A | 144 | 1 | 182.25 | 0.3 | 20 | 0.5 | 0.50 |
| A | 144 | 1 | 182.25 | 0.4 | 20 | 0.5 | 0.50 |
| B | 36 | 1 | 56.25 | 0.1 | 20 | 0.5 | 0.65 |
| B | 36 | 1 | 56.25 | 0.2 | 20 | 0.5 | 0.65 |
| B | 36 | 1 | 56.25 | 0.3 | 20 | 0.5 | 0.65 |
| B | 36 | 1 | 56.25 | 0.4 | 20 | 0.5 | 0.65 |
| C | 36 | 1 | 56.25 | 0.1 | 50 | 0.5 | 0.65 |
| C | 36 | 1 | 56.25 | 0.2 | 50 | 0.5 | 0.65 |
| C | 36 | 1 | 56.25 | 0.3 | 50 | 0.5 | 0.65 |
| C | 36 | 1 | 56.25 | 0.4 | 50 | 0.5 | 0.65 |

| Low density (N=20; J=12x12) | | | | | | | | | | |
| --- | --- | --- | --- | --- | --- | --- | --- | --- | --- | --- |
|  | nind | | captures ID | | recaptures | | spatial recaptures | | Counts | |
| ID | mean | 95% IC | mean | 95% IC | mean | 95% IC | mean | 95% IC | mean | 95% IC |
| 0.1 | 7 | 4-10 | 9 | 4-14 | 2 | 0-5 | 1 | 0-4 | 115 | 87-143 |
| 0.2 | 11 | 6-15 | 18 | 12-25 | 7 | 3-12 | 4 | 2-8 | 106 | 80-131 |
| 0.3 | 13 | 8-16 | 27 | 20-35 | 15 | 10-21 | 8 | 3-13 | 96 | 72-121 |
| 0.4 | 16 | 12-19 | 40 | 29-51 | 24 | 15-33 | 12 | 7-19 | 84 | 63-103 |
| Medium density (N=20; J=6x6) | | | | | | | | | | |
|  | nind | | captures ID | | recaptures | | spatial recaptures | | Counts | |
| ID | mean | 95% IC | mean | 95% IC | mean | 95% IC | mean | 95% IC | mean | 95% IC |
| 0.1 | 7 | 4-11 | 10 | 4-17 | 2 | 0-6 | 1 | 0-4 | 120 | 80-160 |
| 0.2 | 11 | 7-15 | 20 | 10-32 | 9 | 3-18 | 5 | 1-9 | 110 | 75-144 |
| 0.3 | 12 | 8-16 | 29 | 18-44 | 17 | 8-30 | 8 | 3-14 | 100 | 66-131 |
| 0.4 | 15 | 11-19 | 42 | 25-64 | 27 | 14-47 | 12 | 5-20 | 87 | 59-113 |
| High density (N=50; J=6x6) | | | | | | | | | | |
|  | nind | | captures ID | | recaptures | | spatial recaptures | | Counts | |
| ID | mean | 95% IC | mean | 95% IC | mean | 95% IC | mean | 95% IC | mean | 95% IC |
| 0.1 | 20 | 16-25 | 31 | 24-40 | 10 | 6-16 | 6 | 3-10 | 295 | 248-359 |
| 0.2 | 30 | 25-35 | 64 | 52-80 | 34 | 24-47 | 17 | 10-25 | 262 | 221-320 |
| 0.3 | 35 | 28-40 | 92 | 74-115 | 57 | 42-77 | 27 | 17-38 | 234 | 199-284 |
| 0.4 | 39 | 32-43 | 122 | 98-149 | 83 | 63-108 | 36 | 24-28 | 205 | 175-246 |

## Table 2. Information about the scenarios simulated (mean and 95% confidence interval): *nind*, number of individuals detected; *captures ID*, capture events with identification; *recaptures*, recaptures with ID; *spatial recaptures*, individuals captured in more than 1 locations; *counts*, capture events without ID.
