# Supplementary material for: Spatial capture–recapture with random thinning for unidentified encounters
Source: Ecol Evol. 2020 Dec 8;11(3):1187–98. doi: 10.1002/ece3.7091 (PMC7863675; doi:10.1002/ece3.7091)
Supplement: Supplementary file 4 — Appendix S4 [file ECE3-11-1187-s004.docx]

Appendix 4: Simulations results

Table 1. Posterior mean. Three scenarios (A, B and C) simulated with 144, 36 and 36 traps. In each scenario we conducted 100 simulations for random thinning-SCR (RT-SCR) and standard SCR (SCR). *ID*, identification rate; *N*, population size simulated; $\sigma$; half-normal parameter;$\lambda_{0}$, baseline detection rate. We evaluated the simulations using the relative bias (Rel. bias) and Root Mean Square Error (RMSE) for all parameters.

|  |  |  | **Scenario A: *J*=12x12** | | | **Scenario B:**  ***J*=6x6** | | | **Scenario C:**  ***J*=6x6** | | |
| --- | --- | --- | --- | --- | --- | --- | --- | --- | --- | --- | --- |
|  |  |  | $N=20; d\approx0.1$ | | | $N=20; d\approx0.4$ | | | $N=50; d\approx0.9$ | | |
| ID | Model | Evaluation | N | $\sigma$ | $\lambda_{0}$ | N | $\sigma$ | $\lambda_{0}$ | N | $\sigma$ | $\lambda_{0}$ |
| 10 | RT-SCR | Rel. bias | 0.053 | -0.009 | 0.042 | 0.264 | 0.003 | 0.031 | 0.022 | 0.030 | -0.208 |
|  |  | RMSE | 3.481 | 0.041 | 0.081 | 10.439 | 0.074 | 0.182 | 11.319 | 0.056 | 0.145 |
|  | SCR | Rel. bias | 0.659 | -0.047 | -0.781 | 0.543 | 0.141 | -0.796 | 0.026 | -0.007 | -0.875 |
|  |  | RMSE | 16.913 | 0.203 | 0.401 | 14.993 | 0.260 | 0.404 | 11.601 | 0.076 | 0.570 |
| 20 | RT-SCR | Rel. bias | 0.032 | -0.008 | 0.050 | 0.090 | 0.020 | 0.024 | -0.039 | 0.028 | -0.210 |
|  |  | RMSE | 2.756 | 0.037 | 0.083 | 5.744 | 0.058 | 0.165 | 6.149 | 0.045 | 0.143 |
|  | SCR | Rel. bias | 0.226 | -0.001 | -0.805 | 0.148 | 0.037 | -0.746 | -0.030 | 0.003 | -0.775 |
|  |  | RMSE | 8.798 | 0.106 | 0.405 | 7.759 | 0.118 | 0.378 | 6.096 | 0.053 | 0.505 |
| 30 | RT-SCR | Rel. bias | -0.007 | 0.007 | 0.051 | -0.033 | 0.029 | 0.078 | -0.024 | 0.019 | -0.216 |
|  |  | RMSE | 2.288 | 0.037 | 0.081 | 3.593 | 0.048 | 0.154 | 5.071 | 0.037 | 0.145 |
|  | SCR | Rel. bias | -0.083 | 0.000 | -0.694 | -0.060 | -0.012 | -0.596 | -0.029 | 0.006 | -0.692 |
|  |  | RMSE | 3.932 | 0.074 | 0.352 | 4.099 | 0.068 | 0.307 | 5.113 | 0.043 | 0.451 |
| 40 | RT-SCR | Rel. bias | 0.019 | -0.001 | 0.034 | 0.025 | 0.012 | 0.033 | 0.019 | 0.005 | -0.227 |
|  |  | RMSE | 1.903 | 0.033 | 0.074 | 2.983 | 0.042 | 0.140 | 4.247 | 0.028 | 0.150 |
|  | SCR | Rel. bias | 0.034 | 0.017 | -0.663 | 0.034 | 0.014 | -0.647 | 0.018 | 0.001 | -0.615 |
|  |  | RMSE | 2.692 | 0.054 | 0.333 | 3.262 | 0.059 | 0.426 | 4.188 | 0.033 | 0.401 |

Table 2. Posterior median. Three scenarios (A, B and C) simulated with 144, 36 and 36 traps. In each scenario we conducted 100 simulations for random thinning-SCR (RT-SCR) and standard SCR (SCR). *ID*, identification rate; *N*, population size simulated; $\sigma$; half-normal scale parameter;$\lambda_{0}$, baseline detection rate. We evaluated the simulations using the relative bias (Rel. bias) and Root Mean Square Error (RMSE) for all parameters.

|  |  |  | **Scenario A: *J*=12x12** | | | **Scenario B:**  ***J*=6x6** | | | **Scenario C:**  ***J*=6x6** | | |
| --- | --- | --- | --- | --- | --- | --- | --- | --- | --- | --- | --- |
|  |  |  | $N=20; d\approx0.1$ | | | $N=20; d\approx0.4$ | | | $N=50; d\approx0.9$ | | |
| ID | Model | Evaluation | N | $\sigma$ | $\lambda_{0}$ | N | $\sigma$ | $\lambda_{0}$ | N | $\sigma$ | $\lambda_{0}$ |
| 10 | RT-SCR | Rel. bias | 0.027 | -0.011 | 0.029 | 0.151 | -0.003 | 0.074 | -0.020 | 0.023 | -0.213 |
|  |  | RMSE | 3.276 | 0.041 | 0.079 | 9.231 | 0.074 | 0.179 | 10.600 | 0.053 | 0.148 |
|  | SCR | Rel. bias | 0.510 | -0.188 | -0.866 | 0.394 | -0.080 | -0.865 | -0.030 | -0.035 | -0.884 |
|  |  | RMSE | 15.621 | 0.194 | 0.436 | 13.644 | 0.186 | 0.435 | 10.726 | 0.075 | 0.575 |
| 20 | RT-SCR | Rel. bias | 0.014 | -0.012 | 0.036 | 0.041 | 0.013 | 0.002 | -0.051 | 0.023 | -0.213 |
|  |  | RMSE | 2.707 | 0.037 | 0.079 | 5.151 | 0.056 | 0.162 | 6.290 | 0.044 | 0.145 |
|  | SCR | Rel. bias | 0.132 | -0.039 | -0.827 | 0.067 | -0.007 | -0.773 | -0.045 | -0.004 | -0.781 |
|  |  | RMSE | 7.416 | 0.101 | 0.416 | 6.719 | 0.106 | 0.391 | 6.263 | 0.052 | 0.508 |
| 30 | RT-SCR | Rel. bias | -0.025 | 0.004 | 0.039 | -0.059 | 0.023 | 0.058 | -0.031 | 0.016 | -0.219 |
|  |  | RMSE | 2.343 | 0.037 | 0.078 | 3.672 | 0.047 | 0.148 | 5.150 | 0.036 | 0.146 |
|  | SCR | Rel. bias | -0.116 | -0.016 | -0.713 | -0.088 | -0.029 | -0.619 | -0.038 | 0.001 | -0.696 |
|  |  | RMSE | 4.132 | 0.073 | 0.361 | 4.188 | 0.067 | 0.317 | 5.220 | 0.043 | 0.454 |
| 40 | RT-SCR | Rel. bias | 0.007 | -0.004 | 0.023 | 0.010 | 0.007 | 0.015 | 0.012 | 0.002 | -0.229 |
|  |  | RMSE | 1.881 | 0.032 | 0.072 | 2.980 | 0.041 | 0.136 | 4.180 | 0.028 | 0.152 |
|  | SCR | Rel. bias | 0.011 | 0.007 | -0.675 | 0.011 | 0.004 | -0.659 | 0.011 | -0.002 | -0.619 |
|  |  | RMSE | 2.619 | 0.052 | 0.339 | 3.140 | 0.057 | 0.433 | 4.106 | 0.032 | 0.404 |

Table 3. Posterior mode. Three scenarios (A, B and C) simulated with 144, 36 and 36 traps. In each scenario we conducted 100 simulations for random thinning-SCR (RT-SCR) and standard SCR (SCR). *ID*, identification rate; *N*, population size simulated; $\sigma$; half-normal scale parameter;$\lambda_{0}$, baseline detection rate. We evaluated the simulations using the relative bias (Rel. bias) and Root Mean Square Error (RMSE) for all parameters.

|  |  |  | **Scenario A: *J*=12x12** | | | **Scenario B:**  ***J*=6x6** | | | **Scenario C:**  ***J*=6x6** | | |
| --- | --- | --- | --- | --- | --- | --- | --- | --- | --- | --- | --- |
|  |  |  | $N=20; d\approx0.1$ | | | $N=20; d\approx0.4$ | | | $N=50; d\approx0.9$ | | |
| ID | Model | Evaluation | N | $\sigma$ | $\lambda_{0}$ | N | $\sigma$ | $\lambda_{0}$ | N | $\sigma$ | $\lambda_{0}$ |
| 10 | RT-SCR | Rel. bias | -0.020 | -0.015 | 0.006 | -0.030 | -0.011 | -0.048 | -0.099 | 0.009 | -0.224 |
|  |  | RMSE | 3.215 | 0.042 | 0.077 | 7.804 | 0.074 | 0.179 | 10.378 | 0.050 | 0.154 |
|  | SCR | Rel. bias | 0.082 | -0.328 | -0.931 | -0.015 | -0.252 | -0.923 | -0.127 | -0.081 | -0.900 |
|  |  | RMSE | 13.682 | 0.219 | 0.466 | 9.943 | 0.192 | 0.462 | 11.039 | 0.080 | 0.586 |
| 20 | RT-SCR | Rel. bias | -0.024 | -0.016 | 0.015 | -0.048 | 0.000 | -0.036 | -0.073 | 0.015 | -0.219 |
|  |  | RMSE | 2.676 | 0.037 | 0.076 | 4.443 | 0.054 | 0.159 | 6.713 | 0.043 | 0.149 |
|  | SCR | Rel. bias | -0.023 | -0.095 | -0.862 | -0.067 | -0.069 | -0.813 | -0.072 | -0.020 | -0.792 |
|  |  | RMSE | 5.730 | 0.103 | 0.433 | 5.525 | 0.102 | 0.410 | 6.772 | 0.052 | 0.515 |
| 30 | RT-SCR | Rel. bias | -0.050 | -0.001 | 0.017 | -0.103 | 0.012 | 0.022 | -0.046 | 0.011 | -0.222 |
|  |  | RMSE | 2.496 | 0.037 | 0.075 | 3.823 | 0.044 | 0.141 | 5.348 | 0.035 | 0.149 |
|  | SCR | Rel. bias | -0.167 | -0.044 | -0.745 | -0.145 | -0.057 | -0.657 | -0.055 | -0.007 | -0.705 |
|  |  | RMSE | 4.634 | 0.073 | 0.375 | 4.629 | 0.069 | 0.334 | 5.598 | 0.042 | 0.460 |
| 40 | RT-SCR | Rel. bias | -0.018 | -0.009 | 0.000 | -0.025 | -0.002 | -0.016 | 0.001 | -0.002 | -0.232 |
|  |  | RMSE | 1.911 | 0.033 | 0.070 | 2.912 | 0.040 | 0.132 | 4.108 | 0.028 | 0.153 |
|  | SCR | Rel. bias | -0.030 | -0.012 | -0.697 | -0.028 | -0.014 | -0.681 | -0.003 | -0.008 | -0.626 |
|  |  | RMSE | 2.625 | 0.052 | 0.350 | 3.145 | 0.055 | 0.447 | 4.048 | 0.032 | 0.409 |

Table 4. Population size estimate for simulations (low, medium and high density and 10, 20, 30 and 40% ID, using random thinning-SCR [RT-SCR] and standard SCR). Coverage rates for the 95% highest posterior density (HPD) intervals.

| ID | Model | Low density | Medium density | High density |
| --- | --- | --- | --- | --- |
| 10 | RT-SCR | 0.97 | 0.96 | 0.99 |
|  | SCR | 0.99 | 0.96 | 0.99 |
| 20 | RT-SCR | 0.98 | 0.96 | 0.90 |
|  | SCR | 0.95 | 0.95 | 0.97 |
| 30 | RT-SCR | 0.96 | 0.96 | 0.97 |
|  | SCR | 0.91 | 0.92 | 0.96 |
| 40 | RT-SCR | 0.96 | 0.96 | 0.97 |
|  | SCR | 0.99 | 0.95 | 0.96 |
