# Supplementary material for: Spatial capture–recapture with random thinning for unidentified encounters
Source: Ecol Evol. 2020 Dec 8;11(3):1187–98. doi: 10.1002/ece3.7091 (PMC7863675; doi:10.1002/ece3.7091)
Supplement: Supplementary file 5 — Appendix S5 [file ECE3-11-1187-s005.docx]

Appendix 5: Comparison with multiple observation processes (MOP) from Tourani et al (2020)

In this Appendix, we will describe the process leading to dependence between identified and unidentified detections of the same type collected in the same locations and show that the model of Tourani et al. (2020) relies on the same Poisson encounter model assumption we use to ensure independence between detections of each type. Further, we will show that the random thinning model allows more flexibility to accommodate other encounter models while maintaining independence between the identified and unidentified detections.

1. Decomposing site visitation and detection

We are specifically interested in the spatial capture-recapture (SCR) observation model, since the ecological process models of the random thinning and Tourani models are identical. The SCR observation model describes how we observe individual detections or counts as a function of the distance between an activity center and a trap governed by a detection function. This observation process can be broken down into at least two subprocesses–site visitation and detection conditional on site visitation. We are interested in this decomposition because the site visitation process leads to dependence between individual detections when more than one detection can be made on a single site visit as is the case for scats, hair samples, photos, and other non-invasive sampling methods.

In practice, site visitation and detection are pooled into a single process because there is generally no information to separate them. To see this, consider a site visitation process where a detection function determines the probability of site visitation:

$$p_{ij}=p_{0}exp\left( \frac{{-d}_{ij}^{2}}{{2\sigma}^{2}} \right)$$

where $p_{ij}$ the probability individual $i$ visits site $j$ on a single occasion, $d_{ij}$ is the distance between the activity center of individual $i$ and trap $j$, and $\sigma$ is the site visitation spatial scale parameter. We can then assume $y_{ijk}$, the site visitation events for individual $i$ at trap $j$ on occasion$k$ are Bernoulli:

$$y_{ijk}\sim Bernoulli(p_{ij})$$

Next, consider that conditional on site visitation, $y_{ijk}=1$, individuals have probability $q$ of being detected. We can also assume detection conditional on site visitation is a Bernoulli random variable. Then, the probability individual $i$ will visit site $j$ on a single occasion and be detected is$p_{ij}^{'}=p_{ij}q$. Because both processes are Bernoulli, we can combine them into a single Bernoulli process with a single detection function.

$$p_{ij}^{'}=p_{0}^{'}exp\left( \frac{-d_{ij}^{2}}{{2\sigma}^{2}} \right)$$

$$y_{ijk}^{'}\sim Bernoulli\left( p_{ij}^{'} \right)$$

where $p_{0}^{'}=p_{0}q$, and $y_{ijk}^{'}$ is 1 if individual $i$ visited site$j$ on occasion $k$ and was detected and 0 otherwise. This result is an example of the general result for the distribution of a Binomial random variable conditioned on the outcome of another Binomial random variable (Pinsky and Karlin 2010, p. 47). In practice, we estimate $p_{0}^{'}$, the combined probability of site visitation and detection, again, because these typically cannot be separated with the data we collect.

Now, consider we place two traps of the same type at each site, both with detection probability $q$ conditional on site visitation. Further, we might assume that these detection processes are independent of one another–conditional on site visitation, the detection events are independent. We might then treat the detections from each trap (not conditional on site visitation) as independent, assuming:

$$y_{ijk}^{'1}\sim Bernoulli\left( p_{ij}^{'} \right)$$

$$y_{ijk}^{'2}\sim Bernoulli\left( p_{ij}^{'} \right)$$

where “1” and “2” distinguish the data collected by each trap. However, in this case, $y_{ijk}^{1}$ and $y_{ijk}^{2}$ are not independent because they both depend on the outcome of the single site visitation random variable $y_{ijk}$. If we observe $y_{ijk}^{'1}=0$, we are more likely to observe $y_{ijk}^{'2}=0$ because at least some of the 0 detection events in an SCR survey are due to the failure of individuals to visit the site. Similarly, if we observe $y_{ijk}^{'1}=1$, $y_{ijk}^{'2}=1$ is more likely than if we observed $y_{ijk}^{'1}=0$ because the detection in trap 1 implies that the individual visited the site on that occasion.

2. The MOP Model - Description and Lack of Independence

Next, consider the structure of the Tourani observation model. They consider an observation model where individuals may be detected across sites with $M$ trap types, which are not co-located, and occasions. Each of the $M$ trap types produce individual identities for detection events with probability $1-\alpha^{m}$ and no individual identities with probability $\alpha^{m}$. For example, these trap types may be discretized scat transects or camera traps, both of which obtain detections that may be identified to individual or not. The probability of detecting individual $i$at trap $j$ on occasion $k$ for trap type $m$ is then:

$$p_{ij}^{m}=p_{0}\exp\left( \frac{-d_{ij}^{m^{2}}}{{2\sigma}^{2}} \right)$$

where $d_{ij}^{m}$ is the distance between individual $i$ and trap $j$ of type $m$. From here, two observation models are used, one for the identified detections and a second for the unidentified detections. Tourani et al. (2020) treat the unidentified detections as occupancy data similar to Chandler & Clark (2014) by reducing the detection data to presence/absence data for each combination of trap and occasion. The probability any individual is detected at trap $j$ of type $m$ is:i9

$$p_{.j}^{m}=1-\prod_{i=1}^{N} (1-p_{ij}^{m}\left( 1-\alpha^{m} \right))$$

where $N$ is individual abundance. Then, the observation models for the identified and unidentified detections are:

$$y_{ijk}^{m}\sim Bernoulli\left( p_{ij}^{m}\alpha^{m} \right)$$

$$y_{.jk}^{m}\sim Bernoulli\left( p_{.j}^{m} \right)$$

Here, we note that $y_{ijk}^{m}$ and $y_{.jk}^{m}$ are not generally independent because they are both conditional on the same site visitation events as argued above–identified and unidentified samples can be deposited on the same site visit. This lack of independence, in principle, can cause bias and an underestimation of the parameter posterior variances as shown by Clare et al. (2017). In practice, the effect on inference will depend on the magnitude of the correlation between detections of each type at the same trap-occasion.

One caveat here is that the detections of identified and unidentified samples could be regarded as independent if there were two site visitation processes, one where individuals visit a site and may only leave identified detections, and a second where individuals visit a site and may only leave unidentified detections. However, we find this implausible for detectors that collect DNA or photos—in general, individuals will be able to leave either identified or unidentified detections on the same site visit.

3. The MOP Model – A Special Case for Independence

Here, we will note that there is a more plausible special case where the identified and unidentified data types at co-located traps can be regarded as independent–when the data generating observation model is Poisson with individual identities thinned at the sample level as we have assumed for the random thinning model. Without considering multiple trap types, we assume the baseline detection rate is:

$$\lambda_{ij}=\lambda_{0}\exp\left( \frac{-d_{ij}^{2}}{2\sigma^{2}} \right)$$

The true count detections are then:

$$y_{ijk}^{true}\sim Poisson(\lambda_{ij})$$

and we thin the true counts directly:

$$y_{ijk}^{ID}\sim Binomial(y_{ijk}^{true},\theta)$$

$$y_{ijk}^{noID}=y_{ijk}^{true}-y_{ijk}^{ID}$$

Our approach is to use $y_{ijk}^{ID}$ and $y_{ijk}^{noID}$, summed across individuals to probabilistically reconstruct the single count encounter history $y_{ijk}^{true}$. However, because of the Poisson assumption for detections and the fixed thinning rate, the identified and latent identity unidentified counts are distributed as *independent* Poisson random variables with encounter rates $\theta\lambda_{ij}$ and $(1-\theta)\lambda_{ij}$, respectively, despite being co-located. This follows from general results for thinned Poisson processes which can be found in Chiu et al. (2013) and elsewhere. To our knowledge, this result does not hold for any other count distribution, for example the negative binomial, and does not hold when the thinning rate varies across samples. After thinning the Poisson-distributed detections to identified and unidentified count data sets, the identified count data set can be converted to Bernoulli data and the unidentified count data set can be converted into presence-absence data leaving us with the observation model of Tourani et al. (2020).

Therefore, the model of Tourani does not have a lack of independence between identified and unidentified detections when the data generating observation model is Poisson and when all samples are thinned of individual identities at the same rate. When the Poisson assumption is true, the random thinning model will produce more precise parameter estimates because the count data are more informative than the reduced Bernoulli and presence/absence detections. Further, we can replace the Poisson assumption in our model with other count distributions and allow the thinning rate to vary across individuals and samples, providing more flexibility without violating independence. The random thinning model can also be extended to accommodate a behavioral response to capture, which requires both probabilitistically reconstructing the true encounter history and the true individual by trap by occasion behavioral states (i.e., when individuals are exposed to first and subsequent capture).

Finally, we note that the Tourani model assumes that the multiple trap types, for example, scat surveys and camera traps, are not co-located to ensure independence. If multiple trap types are co-located, the model of Clare et al. (2017) is appropriate, but requires individual identities for all data types. This model has been extended to accommodate unknown individual identities for one of two trap types (e.g., identified scats, unidentified photos; Sun, 2019).

References

Chandler, R.B. & Clark, J.D. (2014). Spatially explicit integrated population models. *Methods in Ecology and Evolution*, 5, 1351–1360.

Chiu, S.N., Stoyan, D., Kendall,W.S. & Mecke, J. (2013). *Stochastic geometry and its applications*. John Wiley & Sons.

Clare, J., McKinney, S.T., DePue, J.E. & Loftin, C.S. (2017). Pairing field methods to improve inference in wildlife surveys while accommodating detection covariance. *Ecological applications*, 27, 2031–2047.

Pinsky, M. and Karlin, S. (2010). An introduction to stochastic modeling. Academic press.

Sun, C. (2019). *Identifying landscape-wide spatial heterogeneity in population density and genetic structure of American black bear (Ursus americanus) in New York and the northeastern United States*. Ph.D. thesis, Cornell University.

Tourani, M., Dupont, P., Nawaz, M.A. & Bischof, R. (2020). Multiple observation processes in spatial capture–recapture models: How much do we gain? *Ecology*, p. e03030.
